# Room-Temperature Ammonia Sensor Based on ZnO Nanorods Deposited on ST-Cut Quartz Surface Acoustic Wave Devices

**DOI:** 10.3390/s17051142

**Published:** 2017-05-17

**Authors:** Wei Li, Yuanjun Guo, Yongliang Tang, Xiaotao Zu, Jinyi Ma, Lu Wang, Yong Qing Fu

**Affiliations:** 1 School of Physical Electronics, University of Electronic Science and Technology of China, Chengdu 610054, China; 201421040126@std.uestc.edu.cn (W.L.); 201311040302@std.uestc.edu.cn (Y.T.); xtzu@uestc.edu.cn (X.Z.); 2 Sichuan Institute of Piezoelectric and Acousto-Optic Technology, Chongqing 400060, China; ma_jinyi163@163.com (J.M.); wanglu720@163.com (L.W.); 3 Faculty of Engineering & Environment, University of Northumbria, Newcastle upon Tyne NE1 8ST, UK

**Keywords:** NH_3_ sensor, surface acoustic wave (SAW) device, ZnO nanorods, seed layer-free growth

## Abstract

Using a seed layer-free hydrothermal method, ZnO nanorods (NRs) were deposited on ST-cut quartz surface acoustic wave (SAW) devices for ammonia sensing at room temperature. For a comparison, a ZnO film layer with a thickness of 30 nm was also coated onto an ST-cut quartz SAW device using a sol-gel and spin-coating technique. The ammonia sensing results showed that the sensitivity, repeatability and stability of the ZnO NR-coated SAW device were superior to those of the ZnO film-coated SAW device due to the large surface-to-volume ratio of the ZnO NRs.

## 1. Introduction

With rapid economic growth and the development of modern industry, there are significant negative impacts on people’s health from pollution of the air, water, and soil [1]. Ammonia is one of the most dangerous and potentially explosive industrial gases [2,3], and the detection of ammonia is of great importance for the safety of industry and clinical diagnostics. Among different kinds of ammonia sensors, such as semiconductor sensors [4,5,6] and andelectrochemical sensors [7], surface acoustic wave (SAW) sensors have many superior advantages including high speed, small size, high sensitivity, low cost, good reliability, and wireless ability [8,9,10,11,12,13,14]. The key part of a SAW gas sensor is the sensitive layer, whose conductivity or mass change will cause a frequency change of the SAW device [15].

ZnO is a useful semiconductor material for various practical applications due to its desirable electronic, optical and chemical properties [16]. Recently, ZnO nanorods (NRs) have been demonstrated to have wide applications in solar cells, biosensors, nanogenerators, ultraviolet detectors, and humidity sensing, etc. [17,18,19,20,21]. The hydrothermal method is considered a promising technique to synthesize ZnO nanorods (NRs) owing to its low cost and ease of growth. However, a conventional hydrothermal method has two process steps [22,23,24,25,26,27,28]: (1) fabrication of the seed layer; and (2) growth of ZnO NRs on this seed layer. This two-step growth method is relatively complicated and time-consuming for growing ZnO NRs. On the other hand, temperature is one of the key parameters that affect the stability of SAW devices due to its influences on both external and internal stresses [29]. ST-cut quartz has an excellent temperature stability near room temperature in all the piezoelectric materials [30,31], which makes it suitable for SAW sensing applications without external temperature compensation. To our knowledge, room temperature ammonia sensors based on ZnO NRs deposited on ST-cut quartz SAW devices using a seed layer-free hydrothermal method has not yet been reported.

In this work, ZnO NRs were deposited on ST-cut quartz based SAW devices via a seed layer-free hydrothermal method, which is easier and faster than the traditional hydrothermal process, and their ammonia sensing performance was investigated. For a comparison, an ammonia sensor based on a ZnO nano-layer thick film deposited on an ST-cut quartz substrate was fabricated and characterized to compare with those from the ZnO NRs quartz SAW devices.

## 2. Experimental

An ST-cut quartz (42°75′) was used as the substrate (with a dimension of 12 mm × 3 mm × 0.5 mm) of the SAW device, in which the SAW propagation velocity along the direction perpendicular to the crystallographic *x*-axis (90°-rotated) is 3158 m/s. The interdigital transducers (IDTs) of the SAW device were fabricated with 200-nm thick aluminum and consisted of 30 pairs of fingers with a wavelength of 16 μm using conventional photolithography and lift-off processes. The aperture of the IDTs was 3 mm, and the distance between the IDTs was 4 mm. The SAW resonators had a center frequency of 199.95 MHz, measured using a network analyzer (Hewlett Packard 8714C, Hewlett-Packard, Palo Alto, CA, USA).

Using the low temperature seed-free hydrothermal method, ZnO NRs were deposited on the quartz substrate. The precursor solution contained an equal molar ratio of zinc nitrate hexahydrate (Zn(NO_3_)_2_·6H_2_O) and methenamine (C_6_H_12_N_4_), which were dissolved into 2-methoxyethanol, then the solution was added into a sealed bottle with a 100-mL capacity. The device with its surface facing down and floating on the surface of the solution was kept in the solution of 5 mmol/L at 90 °C for 3 h. During the process, the IDTs of the SAW device wave were covered with a polyimide tape to prevent being etched. After the growth, the device was taken out and cleaned using deionized water to remove the residual and dried at room temperature for further study.

ZnO nanofilms were prepared using sol-gel and spin-coating processes on the surface of the SAW device. The way to prepare the ZnO sol was the same as that described in Reference [8]. Using a spin-coating method with as-prepared ZnO gel at a speed of 3000 rpm for 30 s, a ZnO nanofilm with a thickness of 30 nm was prepared on the surface of the SAW devices. Then, the coated SAW devices were immediately transferred into the furnace to be kept at 300 °C for 10 min, followed by annealing at a temperature of 500 °C for 1 h.

The morphology of the prepared ZnO nanofilm and NRs were characterized using a field-emission scanning electron microscope (FE-SEM, Carl Zeiss 1530 VP, Carl Zeiss Mircoscopy, Thornwood, NY, USA) and an atomic force microscope (AFM, Being technology 5500, Being Nano Instruments LTD., Beijing, China). A Rigaku D/max-2400 X-ray diffractometer (Rigaku, Tokyo, Japan) was applied to characterize the crystallinity of the prepared nanofilm and NRs.

**Figure 1:**
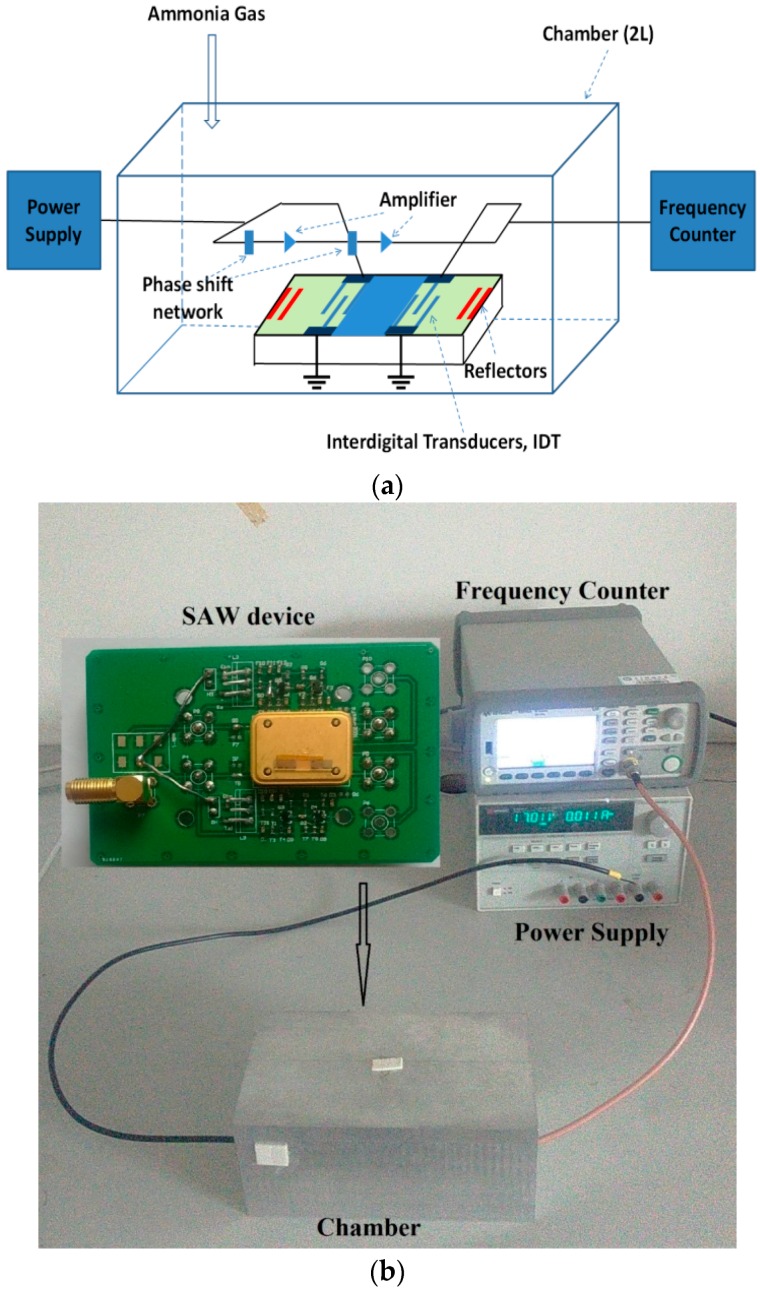
Measurement system of ammonia gas: (**a**) schematic diagram; (**b**) picture.

The SAW sensor consisted of the SAW resonator coated with ZnO nanofilm (or NRs) and the corresponding oscillator circuits. A frequency counter (Agilent 53210, Keysight Technologises(M) Sdn Bhd, Penang, Malaysia) was used to measure the output signal of the SAW sensors. The SAW resonators growth with ZnO NRs had a center frequency of 199.76 MHz, and those with ZnO nanofilm had a center frequency of 199.89 MHz. Figure 1 shows the schematic illustration of the measurement system. The SAW sensor was placed inside a testing chamber with a volume of 2000 mL. The target gas was the standard ammonia gas (2% purity), obtained from the National Institute of Testing Technology of China. During the ammonia test, a dynamic volumetric method was adopted by using a syringe to inject the gas into the testing chamber, and the time to inject ammonia into the chamber was about 10 s. When the response reached an equilibrium condition, the cover of the small chamber was removed to be exposed to the atmosphere in the fume cupboard. The chamber was purged with air before the chamber was covered for the next test. When the frequency of the sensor became stable in its intrinsic frequency, the ammonia gas was injected into the testing chamber again. Ammonia sensing characteristics of the SAW sensor were obtained at various ammonia concentrations at room temperature. In addition, the concentrations (10, 20, 40, 60, 80 and 100 ppm) of ammonia were controlled by adjusting its volumes (1, 2, 4, 6, 8 and 10 mL) injected into the tasting chamber. In all the testing, the temperature and humidity in the chamber were maintained at 25 °C and 25%, respectively.

## 3. Results and Discussion

**Figure 2:**
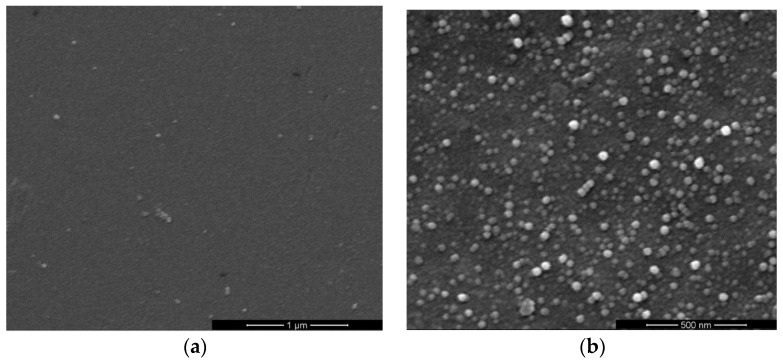
Top-view SEM images of ZnO nanofilms in different scales.

**Figure 3:**
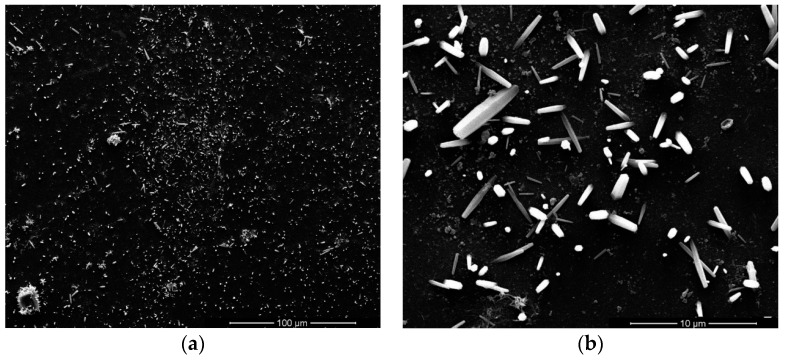
Top-view SEM images of ZnO nanorods (NRs) in different scales.

**Figure 4:**
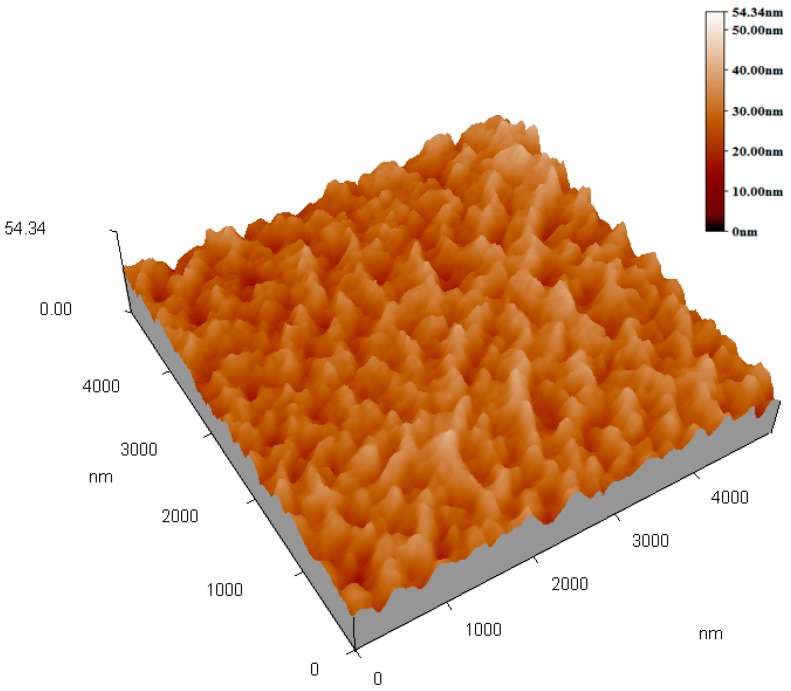
Atomic force microscopy (AFM) image of the ZnO nanofilm.

Figure 2 and Figure 3 show the top-view images of the ZnO nanofilm and NRs, respectively. The surface of the ZnO film is smooth and dense, as shown in Figure 2a,b. The sizes of nanoparticles of the film are in the range of 20–50 nm. Figure 3a shows that the distribution of ZnO NRs is uneven and the growth of ZnO NRs is irregular, and Figure 3b shows the sizes of NRs, whose lengths are in the range of 1–5 μm, and diameters are in the range of about 1–2 μm. The AFM images of the ZnO nanofilm are shown in Figure 4, and the surface roughness of the film is 4.4 nm.

**Figure 5:**
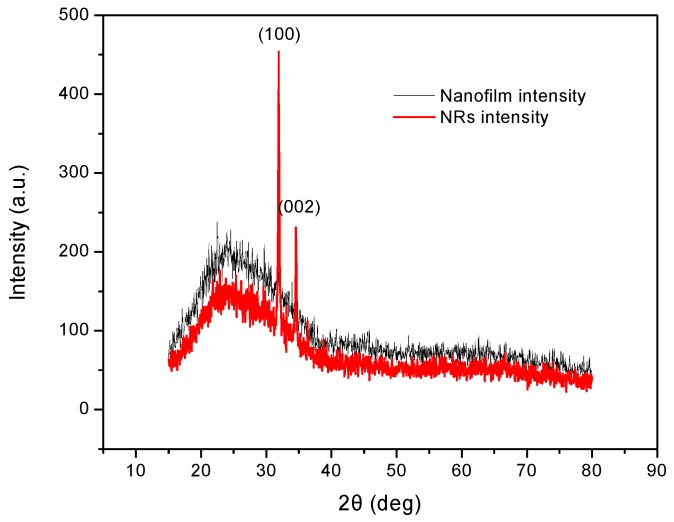
XRD patterns of ZnO nanofilm and NRs.

XRD results of the ZnO nanofilm and NRs are exhibited in Figure 5. The XRD pattern of the ZnO nanofilm shows very weak peaks due to its thin thickness of 30 nm. The thicker line in Figure 5 shows the characteristic peaks of ZnO NRs at 31.77° and 34.42°, which correspond to the (100) and (002) planes of ZnO. The peak at (100) has a relatively higher intensity than that of the peak at (002), which indicates that the ZnO NRs have a preferred oriented orientation along the (100) direction.

**Figure 6:**
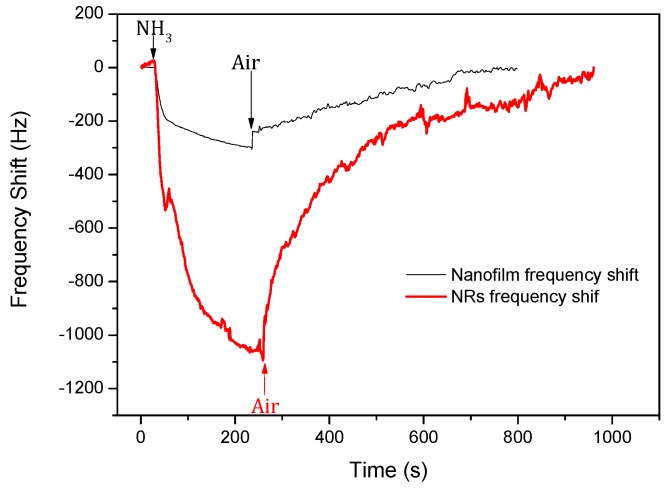
Response of the surface acoustic wave (SAW) sensors with ZnO nanofilm and NRs to 100 ppm ammonia.

Figure 6 shows the responses of the SAW devices with ZnO films and NRs to 100 ppm ammonia with an exposure time of 1000 s. The results show that the two different sensors had obvious responses to the ammonia. The device based on the ZnO nanofilm showed a negative response of −307 Hz. The signal decreased to 90% of its saturated value within 143 s, and then recovered to 90% of the saturated value in 426 s. The device based on ZnO NRs showed a negative response of −1094 Hz, increasing almost 3.6 times as much as that for the sensor with the ZnO nanofilm. As shown in Figure 6, the sensors based on the ZnO NRs responded much faster in the first few seconds, but it took a long time of about 151 s to reach 90% of its saturated level, and the recovery time to 90% of its saturated value was 568 s.

The ammonia gas sensing mechanism can be explained using the Equation (1) [32]:

(1) $$NH_{3}+5O^{-}\to 2NO+3H_{2}O+5e^{-}$$

The oxygen molecules adsorbed on the ZnO layer or NRs formed a depletion layer on the surface due to their strong oxidizability. When the device was exposed to ammonia, the reaction between ammonia molecules and oxygen molecules led to a recombination of electrons and holes, increasing the conductivity of the ZnO layer [33].

Accordingly, the change of SAW velocity (Δ*ν*) is given by [15,34,35]:

(2) $$\frac{\Delta \nu }{\nu_{0}}\approx -\frac{{Κ}^{2}}{2}\frac{\sigma_{s}^{2}}{\sigma_{s}^{2}+\nu_{0}^{2}C_{s}^{2}}$$

where *C_s_* is the surface capacity, σ_s_ is the sheet conductivity, *K* is an electromechanical coefficient, and σ_0_ is the unperturbed SAW velocity. From Equation (2), the conductivity of the sensing layer (σ_s_) varies inversely with the SAW velocity, i.e., the resonant frequency. When the ZnO-coated devices are exposed to the ammonia, the conductivity of the sensing layer (σ_s_) will increase while the SAW velocity will decrease. The ZnO NRs have larger specific surface areas than that of the ZnO film, therefore, the ZnO NRs can absorb more oxygen species and react with more ammonia molecules than ZnO film under the same concentration of ammonia. Therefore, the device with ZnO NRs will have a larger surface conductivity and frequency shift.

**Figure 7:**
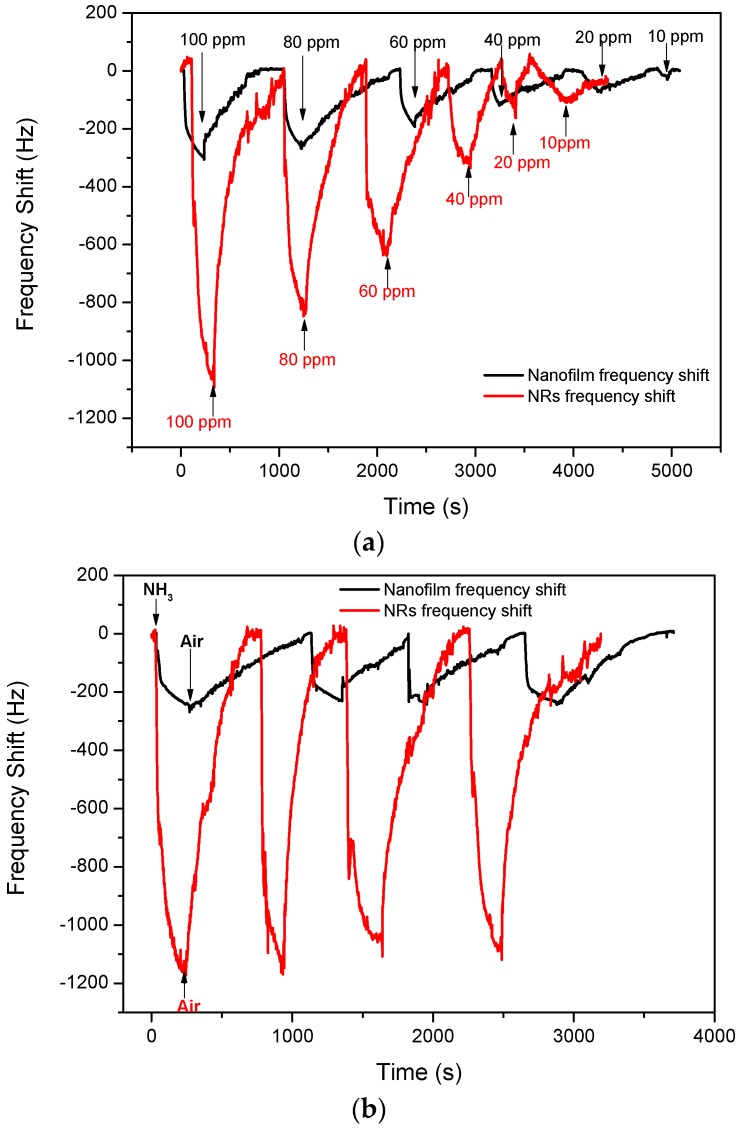

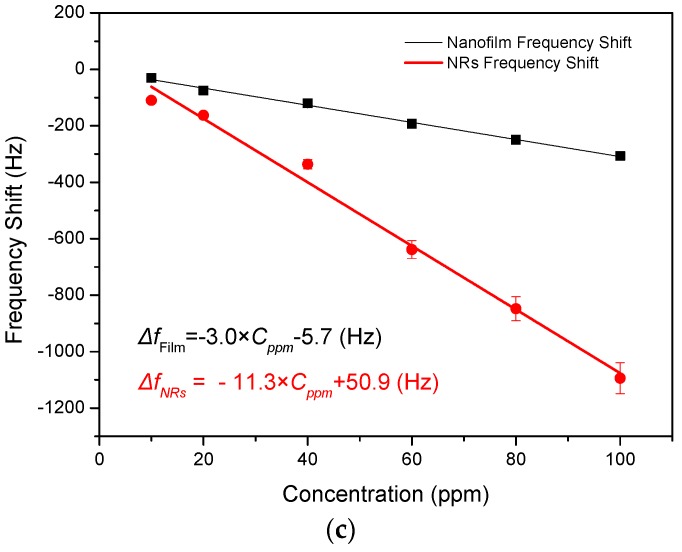
Frequency shifts of SAW devices based on ZnO nanofilm and NRs to (**a**) varying concentrations ammonia; (**b**) 100 ppm ammonia gas for four cycles; and (**c**) frequency shift as a function of ammonia concentration.

Figure 7a shows the frequency shifts of the SAW devices based on ZnO nanofilm and NRs under different concentrations of ammonia gas, and the sensing results are summarized in Table 1. The two sensors showed negative responses to ammonia, and the frequency shift was much smaller but the recovery was much faster at a lower concentration. However, when the sensor using ZnO NRs was exposed to 10 ppm ammonia, the 90% response time and recovery time were much longer than that of 20 ppm. Moreover, when the sensor using ZnO nanofilm was exposed to 100 ppm ammonia, the 90% recovery time was much less than that observed when exposed to 80 ppm. During the recovery process, there were some discontinuous points observed, which probably resulted from the mechanical disturbance when evacuating the ammonia.

The reproducibility of the sensors based on ZnO NRs and films were tested with 100 ppm ammonia gas for four cycles, respectively. As is shown in Figure 7b, the fluctuation of the maximum frequency shift for the SAW sensor with ZnO nanofilm was close to 13%. However, the fluctuation of the maximum frequency shift of the sensor with NRs was less than 10%, and the response and recovery time for each test was nearly the same in the four tests, showing good reproducibility.

In Figure 7c, the frequency shift of the sensor with the ZnO nanofilm as a function of ammonia concentration has a linear relation with a fitted equation as follows:

(3) $$\Delta f=-3.0\times C_{ppm}-5.7\;\left(\mathrm{Hz}\right)$$

where Δ*f* is the frequency shift and C*_ppm_* is the ammonia concentration. Similarly, the relation between the frequency shift and ammonia concentration for the SAW sensor with ZnO NRs is given as:

(4) $$\Delta f=-11.3\times C_{ppm}+50.9\;\left(\mathrm{Hz}\right)$$

From Equations (3) and (4), it is obvious that the slope of Equation (4) is 3.8 times as much as that of Equation (3). This indicates that the response of the sensor with ZnO NRs is 3.8 times as high as that of the sensor based on the ZnO film with the increase in ammonia concentration.

**Figure 8:**
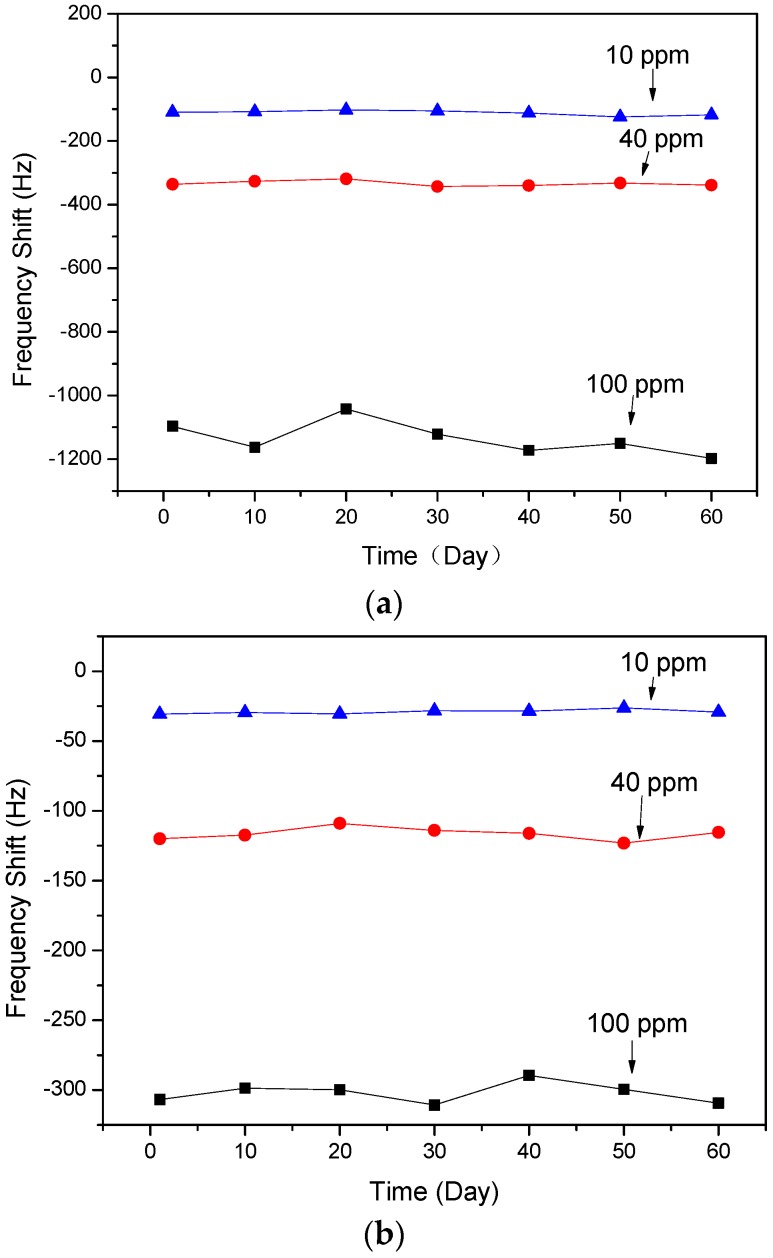
Frequency shifts of SAW devices based on ZnO: (**a**) NRs and (**b**) nanofilm to various concentrations of NH_3_ gas for 60 days.

The stabilities of the two types of SAW sensors were investigated. As showed in Figure 8, the sensors based on ZnO NRs and the nanofilm showed stable responses to certain concentrations of NH_3_ gas for 60 days, respectively.

## 4. Conclusions

ZnO NRs were fabricated via a seed-free hydrothermal method. For a comparison, a ZnO nanofilm was synthetized via a sol-gel and spin-coating method. Room-temperature ammonia sensors based on ST-cut quartz SAW devices using ZnO NRs and nanofilm were investigated. The sensor with the ZnO NRs showed a good stability and reproducibility, and its frequency shift to 100 ppm ammonia gas was −1094 Hz, increasing almost 3.6 times as much as that for the sensor with the ZnO nanofilm. Moreover, when the two SAW devices were exposed to ammonia with lower concentrations, the sensor using the ZnO NRs was much more sensitive than the sensor with the ZnO nanofilm.

## Figures and Tables

**Table 1:** The sensing results of ZnO nanofilm and NRs.

Ammonia Gas Concentration (ppm)	ZnO Nanfilm			ZnO NRs		
	Frequency Shift (Hz)	90% Response Time (s)	90% Recovery Time (s)	Frequency Shift (Hz)	90% Response Time (s)	90% Recovery Time (s)
10	−30	50	34	−110	266	431
20	−75	85	457	−163	117	76
40	−120	94	562	−336	125	233
60	−193	113	583	−638	131	465
80	−269	130	932	−848	140	470
100	−307	143	426	−1094	151	568
